# Sonneradon A Extends Lifespan of *Caenorhabditis elegans* by Modulating Mitochondrial and IIS Signaling Pathways

**DOI:** 10.3390/md20010059

**Published:** 2022-01-08

**Authors:** Shu Jiang, Cui-Ping Jiang, Pei Cao, Yong-Hong Liu, Cheng-Hai Gao, Xiang-Xi Yi

**Affiliations:** Institute of Marine Drugs/School of Pharmaceutical Sciences, Guangxi University of Chinese Medicine, Nanning 530200, China; June.chiang@foxmail.com (S.J.); ping990120@foxmail.com (C.-P.J.); pcao@gxtcmu.edu.cn (P.C.); yonghongliu@scsio.ac.cn (Y.-H.L.)

**Keywords:** antiaging, *Sonneratia apetala*, signaling pathway, *Caenorhabditis elegans*, mutants

## Abstract

Aging is related to the lowered overall functioning and increased risk for various age-related diseases in humans. Sonneradon A (SDA), a new compound first extracted from the edible fruits of mangrove *Sonneratia apetala*, showed remarkable antiaging activity. However, the role of SDA in antiaging remains unclear. In this article, we studied the function of SDA in antiaging by using the animal model *Caenorhabditis elegans*. Results showed that SDA inhibited production of reactive oxygen species (ROS) by 53%, and reduced the accumulation of aging markers such as lipids and lipofuscins. Moreover, SDA also enhanced the innate immune response to *Pseudomonas aeruginosa* infection. Genetic analysis of a series of mutants showed that SDA extended the lifespan of the mutants of *eat-2* and *glp-1*. Together, this effect may be related to the enhanced resistance to oxidative stress via mitochondrial and insulin/insulin-like growth factor-1 signaling (IIS) pathways. The results of this study provided new evidence for an antiaging effect of SDA in *C. elegans,* as well as insights into the implication of antiaging activity of SDA in higher organisms.

## 1. Introduction

*Caenorhabditis elegans* (*C. elegans*) is a powerful experimental system characterized by a short lifespan, the capability to self-reproductive, and ease of cultivation; it has a small and transparent body that is easy to observe, and thus it is commonly used to pre-screen compounds for the research of anticancer, antiaging, and lipid metabolism drugs, or for toxicity studies [1]. Since two-thirds of its genes are homologous to human-disease-related genes, *C. elegans* has been used as a model for studying aging and aging-related diseases [2]. In worms, aging can be detected by the attenuation of exercise and stress ability, cognitive ability, and pharyngeal pump, as well as the accumulation of autofluorescence.

Mangrove is a large group of various salt-tolerant woody trees or shrubs that lives along sheltered coastlines within the tropic or subtropic latitudes. Mangrove plants are usually categorized into two subgroups, true mangrove and semi-mangrove plants. *Sonneratia apetala*, belonging to the family Sonneratiaceae, is an excellent fast-growing true mangrove that is native to India, Bangladesh, Sri Lanka, and other South Asian and Southeast Asian countries [3]. In the 1990s, *S. apetala* was introduced from Sundarban, India to Guangdong, Guangxi and Hainan of China. The ripe edible fruits of *S. apetala* are rich in amino acids, anthocyanins, and other phenolic compounds [4]. In recent years, the search for new molecules with food-additive and nutraceutical applications from marine natural plant extracts has grown significantly. Fruit extracts of *S. apetala* have been found to have numerous beneficial effects on human health, such as antibacterial, antioxidant, and antihepatic lesion activities [4,5]. Studies have shown that acetone, ethanol, methanol, and water extracts from plant leaves and bark had antibacterial effects on *Staphylococcus aureus*, *Shigella flexneri*, *Bacillus licheniformis*, *Bacillus brevis*, *Vibrio cholerae*, *Pseudomonas aeruginosa*, *Bacillus epidermidis*, *Bacillus subtilis,* and *Escherichia coli* at the concentrations of 1.25~5.00 mg/mL [6]. Ethanol fruit extract (250 and 500 mg/kg, p.o.) showed a significant analgesic effect in an acetic-acid-induced writhing model of Swiss albino mice [7]. Some lupeol-derived chalcones extracted from *S. apetala* were found to reduce the levels of total cholesterol, phospholipids, and triacylglycerol in plasma of hyperlipidemia rats, and had antioxidant effects [8]. 

Sonneradon A (SDA, 1A) is a new compound that was extracted from the fruits of *S. apetala* firstly in our previous study [4]. It has been shown to extend the lifespan and healthspan of *C. elegans*. Further, the HSF-1 pathway might be a probable path for antiaging effects by molecular docking. However, the mechanism for its senescence-delaying effect in worms is still unclear. Thus, we studied the mechanisms involved in its bioactivities using the model animal *C. elegans* in this work. Herein, the purpose of this study was to assess the effects of SDA on ROS accumulation, as well as to investigate its effects on fat and autofluorescence accumulation and other indicators related to nematode aging comprehensively, and its underlying mechanism in *C. elegans*. 

## 2. Results

### 2.1. Antioxidant Capacity of SDA in Wild-Type Worms

**Figure 1:**
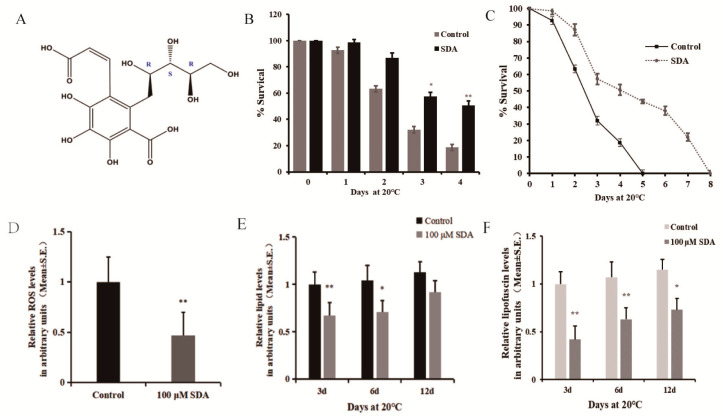
Effect of SDA on oxidative stress in *C. elegans*: (**A**) structure of compound SDA; (**B**) survival time after exposure to paraquat-induced oxidative stress; (**C**) daily survival rate; (**D**) ROS levels; (**E**) relative lipids levels; (**F**) relative autofluorescence levels. The *p*-values were calculated using a two-tailed *t*-test. All data are expressed as mean ± SEM, (n ≥ 30 per group). * *p* < 0.05, ** *p* < 0.01.

Studies have repeatedly shown that antioxidant activity is closely related to the longevity of many organisms [9]. Considering that SDA could increase *C. elegans* lifespan and healthspan, we performed an oxidative stress tolerance assay to determine whether the prosurvival effect of SDA was related to its antioxidant capacity for gaining mechanistic insight. The maximum survival time of the untreated model (control) worms exposed to paraquat oxidative was 5 d, which was extended to 8 d by treatment with 100 μM SDA. On day 4; i.e., the day before the control group reached zero survival, the survival rate of the SDA group was 32% higher than that of the control group (Figure 1B,C).

Moreover, to further validate the link between survival and redox balance in this context, we determined the ROS levels induced by paraquat exposure. The effect of SDA on ROS levels in *C. elegans* under oxidative stress condition was measured using 2′7′-dichlorofluorescin diacetate, and the results are shown in Figure 1D. The results showed that SDA significantly reduced the ROS levels by 53.27%.

*C. elegans* lack mammalian-specific adipocytes, but the neutral fats (triglycerides and cholesterol) stored in its intestinal and subcutaneous cells exist in the form of droplets, which is the main storage form of fat in nematodes. These lipid droplets can be expressed by the fat soluble dye Nile red [10,11]. We stained worms with Nile red to evaluate the effect of SDA on lipid deposition in *C. elegans*, and found that treatment with 100 μM SDA significantly reduced the fat accumulation of *C. elegans* on day 3 (33.0%) and day 6 (31.7%) compared to the control group (*p* < 0.01) (Figure 1E). Moreover, the fluorescence intensity of lipids in worms gradually increased with the test time, and the lipid content of nematodes in the SDA group was 1.89% lower than that in the control group (*p* > 0.05) on day 12. 

Advanced glycation end products (AGEs) are considered a possible cause of aging [12]. Blue fluorescence in nematodes is a possible indicator of AGE accumulation, and *C. elegans* hermaphrodites can serve as a model for investigating the utility of antisenescence interventions that act via the suppression of AGEs production [13]. To further assess the impacts of SDA on *C. elegans* of antiaging, we examined autofluorescence and found that 100 μM SDA treatment delayed the accumulation of autofluorescence as compared with the control group. After 100 μM SDA treatment, the accumulation of autofluorescence decreased by 58.1% compared with the control group on day 3, and the autofluorescence decreased by 44.0% and 41.7% on days 6 and 12, respectively (Figure 1F).

### 2.2. SDA Delay of the Progression of Aging-Related Diseases in C. elegans Models of Alzheimer’s Disease (AD) and Upregulation of the Innate Immune Response 

**Figure 2:**
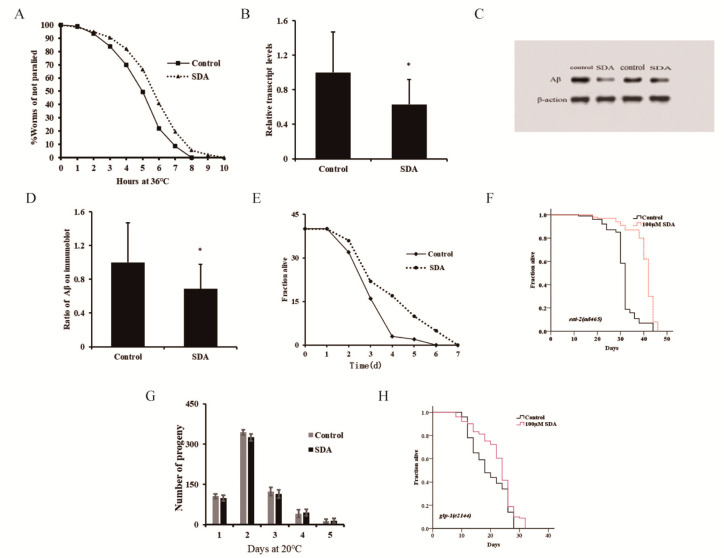
Paralysis rate of CL4176 elegans (**A**). Aβ mRNA levels in *C. elegans* CL4176 (**B**). Aβ protein levels in CL4176 worms (**C**). Ratio of Aβ on immunoblot (**D**). Survival curves of wild-type N2 worms during *P. aeruginosa* infection (**E**). Survival curves of the *eat-2* mutant (**F**). Spawning capacity of *C. elegans* (n = 10) (**G**). Survival curves of the *glp-1* mutant (**H**). The *p*-values were calculated using a two-tailed *t*-test. All data are expressed as mean ± SEM. * *p* < 0.05 compared to control.

Since SDA could extend the lifespan and enhance the antioxidative stress ability of *C. elegans*, we next determined whether it could also ameliorate protein toxicity stress in worms and delay the progression of neurodegenerative diseases. Our results showed that 100 μM SDA could delay the temperature-induced paralysis of CL4176 worms (Figure 2A). After 31 h of culture at 25 °C, the Aβ gene of CL4176 worms in the SDA group was downregulated by 37.09% (Figure 2B), and the protein level decreased by 31.37% compared with the control group (*p* < 0.05) (Figure 2C,D). These findings revealed that SDA has the potential to suppress the toxicity of amyloid plaque.

Many aromatic ring compounds have been shown to possess antioxidant and anti-inflammatory properties [4]. Aging is known to be associated with chronic inflammation, and increasing evidence supports the notion that the chronic inflammation plays a key role in the pathogenesis of several aging-associated diseases. Appropriate immunity is important for extending a healthy lifespan. In this study, we assessed the potential efficacy of SDA in enhancing the innate immune response to *P. aeruginosa* challenge and improving the lifespan of *C. elegans* under the infection condition (Figure 2E). We found that the lifespan of the SDA group was increased by 16.80% (*p* < 0.05).

Pharyngeal pumping can regulate the food intake of elegans, and the change in bulb movement at the end of pharynx can cause dietary restriction (DR) effects. In addition, food intake reduction, which mimics DR in humans, has been shown to increase lifespan in animal studies [14]. Based on this, a lifespan experiment was conducted using the pumping-defective mutant *eat-2 (ad465) II.* of *C. elegans* to determine whether SDA could exert its antiaging activity through DR. Results showed that the survival of nematodes was prolonged by 28.53% (*p* < 0.05) after SDA treatment (Figure 2F), and the impairment of *eat-2* expression had no influence on the SDA-induced effects on the lifespan of wild-type worms. It was thus suggested that SDA may not play a role in delaying senescence through a DR mechanism.

The reproductive ability of *C. elegans* is directly related to the antiaging activity [3]. In the test for the reproductive capacity of SDA-administered nematode worms, we found that the number of eggs laid by nematodes was the largest on day 2 in both the control group and the SDA-administration group, reaching 344 and 325, respectively; there was no difference in the number of offspring larvae of nematodes between the two groups at any time point tested, indicating that SDA treatment had no effect on the daily and total egg production of worms (Figure 2G). When we tested the lifespan of the reproductive-signal-related mutant *glp-1 (e2144) III*, we found that SDA could further extend the worms’ lifespan (Figure 2H). These results suggested that an SDA-induced increase in the lifespan of *C. elegans* may not be related to the reproductive capacity of nematodes.

### 2.3. Potential of SDA to Extend C. elegans’ Lifespan through Insulin/Insulin-like Growth Factor-1 Signaling Pathways

**Figure 3:**
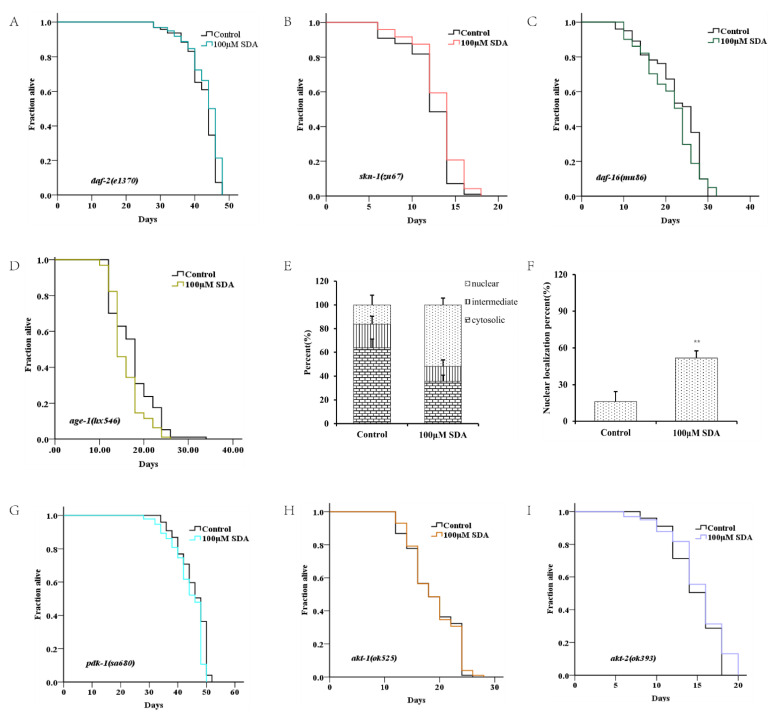

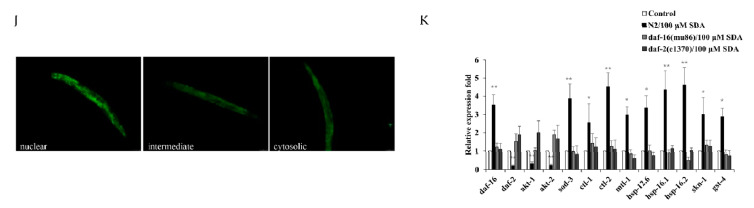
Survival curves for the *daf-2* mutant (**A**). Survival curves for the *daf-16* mutant (**B**). Survival curves for *skn-1* mutants (**C**). Subcellular localization of DAF-16::GFP (**D**). Nuclear translocation of *daf-16* (**E**). Lifespan curves adjusted to Kaplan–Meier estimator for the mutants of *age-1* (**F**), *akt-1* (**G**), *akt-2* (**H**), and *pdk-1* (**I**). Images of the localization of DAF-16::GFP in worms (40×) (**J**). The mRNA level of genes in IIS pathways (**K**). The *p*-values were calculated using a two-tailed t-test. All data are expressed as mean ± SEM, (n ≥ 60). * *p* < 0. 05, ** *p* < 0. 01 compared to control.

The factor DAF-16/FOXO is the central regulator of longevity and oxidative stress tolerance, and the proteins encoded by *daf-16* target genes can protect cells from oxidative stress [15]. To determine how SDA affects lifespan extension and antioxidant stress in nematodes, we first examined expression of the transcription factor *daf-16* by TJ356 worms. The results showed that SDA did not further extend the lifespan of *skn-1* mutants (Figure 3B), suggesting that SDA may be partially dependent on DAF-16. Moreover, with SDA treatment, the DAF-16 accumulation (DAF-16::GFP) in the nucleus of TJ356 worms increased by 38.72% (*p* < 0.01; Figure 3D,E,J).

To determine whether SDA extended *C. elegans’* lifespan by regulating the DAF-16 activity, we measured expression levels of *mtl-1, ctl-1/2, sod-3, hsp-12.6, hsp-16.1*, and *hsp-16.2* of *daf-16*-targeted genes. The results showed that expression levels of these genes were significantly increased in wild-type N2 worms treated with 100 μM SDA compared with the control group (*p* < 0.05), but no such change was detected in *daf-16(mu86) I.* mutants (Figure 3K). Together, these results suggested that SDA may extend the lifespan of *C. elegans* by activating the DAF-16/FOXO pathway.

Like *daf-16*, the SKN-1/NRF2 is also a pivotal oxidative stress response transcription factor for worms. We analyzed the role of skn-1 in the lifespan extension induced by SDA. SDA increased the maximum survival time of the mutant *skn-1(zu67) IV*. by 4.86% (Figure 3C). The mRNA level of gst-4, a target gene of skn-1, was also significantly increased (Figure 3K).

*Daf-16* has been known as an obligatory factor in the insulin/insulin-like growth factor-1 signaling (IIS) pathway. We conducted lifespan tests in *daf-2* mutants to determine whether SDA might interact with the factors in this pathway. The results showed that SDA did not further extend the lifespan of long-lived insulin-like receptor mutant *daf-2 (e1370) III.* (Figure 3A). Moreover, SDA did not alter the expression level of daf-16-targeted genes in *daf-2* mutant worms (Figure 3K).

*DAF-16* in the nucleus interacts with other factors in the IIS pathway known to play an important role in aging, such as *akt-1*, *akt-2*, *age-1* and *skn-1*. Therefore, we investigated the lifespan-extension effect of SDA on the mutants in the IIS pathway. The results showed that the mutants *akt-1(ok525) V.*, *akt-2(ok393) X.*, and *sgk-1(ok538) X.* diminished the effect of SDA on lifespan extension (*p* > 0.05), which suggested that the effect of SDA on lifespan extension might be partially dependent on the *akt-1*, *akt-2*, and *sgk-1* genes. Therefore, we conducted a lifespan test on the loss-of-function mutant *pdk-1(sa680) X.*, given that *pdk-1* regulates the activity of these three factors. Our results showed that SDA did not further extend the longevity of mutant *pdk-1(sa680) X.* Together, these results suggested that SDA may extend the lifespan of worms mainly through regulating IIS pathways.

## 3. Discussion

*S. apetala*, which is native to Bangladesh, is a member of a mangrove community that guards the coast, and plays an important role in maintaining the balance of the earth’s ecosystem [4]. Studies have reported the bioactivity of *S*. *apetala* fruit extracts in rodent models using rats or mice. Recently *C. elegans* has emerged as a good model for mechanistic studies, given its numerous mutants available [16]. We found that SDA extended the lifespan of *C. elegans,* and this effect may be related to the enhanced resistance to oxidative stress via the mitochondrial and IIS pathways. To the best of our knowledge, this is the first study to demonstrate the antiaging activity and mechanism of compounds derived from *S. apetala* in *C. elegans*. 

### 3.1. SDA Reduces Free Radical Production and Enhances Nematode Immunity

Our previous studies have shown that 100 μM SDA can prolong the lifespan of *C. elegans* and enhance their healthspan, while being resistant to heat stress [4]. To advance the research along this line, in the current study, we further investigated the mechanism underlying the antiaging effect of SDA in *C. elegans*. 

With the advancement in age, the accumulation of autofluorescence is pathologically connected with various age-associated disorders. Intestinal fat is involved in energy metabolism, and therefore, the intestine is a main organ for the study of lipid metabolism [1]. In this study, we showed that strong red fluorescence occurred around the intestinal tract of *C. elegans* (Figure S2), which was consistent with the study by Alexandre et al. (2012). Advanced glycation end products (AGEs) were considered as a possible cause of aging [17]. Blue fluorescence in nematodes is a possible indicator of AGEs accumulation, and *C. elegans* hermaphrodites can serve as a model for investigating the utility of antisenescence interventions that act via the suppression of AGEs production. In addition, Komura et al. showed that blue autofluorescence was expected to be a better biomarker for tracing senescence [12]. CL4176 was genetically engineered to express a temperature-inducible human Aβ protein in the body wall muscle of the worm. The Aβ expression is muscle-directed by the use of the myo-3 promoter. When the strain was propagated at 15 °C, there was no expression of Aβ, while upshifting the temperature to 25 °C induced the expression of human Aβ protein, and the worms became paralyzed. The extent of paralysis reflected the toxicity caused by the expression of Aβ.

The exact relationship among autofluorescence, senescence, and lifespan in the worm has remained unclear. Gerstbrein et al. reported that age pigments were valid reporters of the nematode healthspan [18], whereas Coburn et al. reported that blue fluorescence served as a death marker for several hours before and after death in *C. elegans* [19]. It has been suggested that the increases in blue autofluorescence over time observed in populations of aging *C. elegans* might reflect not the aging rate or health state of the population, but the fraction of dead or almost-dead individuals in the sample. To assess the utility of autofluorescence as a biomarker of senescence in *C. elegans*, Komura et al. measured the autofluorescence of individual nematodes using spectrofluorometry. The results showed that blue fluorescence in nematodes was a possible indicator of AGEs accumulation, and that *C. elegans* hermaphrodites can serve as a model for investigating the utility of antisenescence interventions that act via the suppression of AGEs production. In addition, autofluorescence was expected to be a better biomarker for tracing senescence [13]. It has been reported that an increased level of intracellular ROS contributed to the aggregation of autofluorescence [20]. The free radical theory of aging is a widely accepted hypothesis that describes ROS as tremendously reactive molecules that contribute to aging and age-related manifestations [21]. Paraquat is a reagent commonly used to establish an oxidative stress model in nematodes, and it can induce a surge in reactive oxygen radicals in worms, resulting in cell degeneration and necrosis [3]. ROS is a byproduct of normal metabolism; however, its excessive and unbalanced production is known to cause deleterious cellular damage, leading to the oxidative damage of lipids and proteins, a prominent manifestation and mechanism of the aging process [22]. SDA exerts indirect antioxidant potential by the modulation of antioxidant defense systems. 

Once the pathogenic bacteria are deposited in the intestine, they will invade the host cells and even kill nematodes through the process of infection. *C. elegans* usually responds to this situation by avoiding pathogens or activating its inducible innate immune system. *P. aeruginosa* infection is often secondary to immune damage from various causes. Due to its adaptability to genetic manipulation, susceptibility to many human and animal pathogens, and conservative immune signal transduction pathway, *C. elegans* has become a suitable model for studying the innate immunity of human and other animal hosts *C. elegans* protects itself from disease-causing bacteria through its innate immune system [23]. We found that SDA enhanced tolerance to oxidative stress (Figure 1B,C) and reduced intracellular ROS levels (Figure 1D and Figure S1) in wild-type worms, supporting our hypothesis regarding the mitigated ROS-mediated lifespan extension. SDA also improved the innate immune response and resistance to bacterial infection. All these favorable effects of SDA may contribute to its longevity-promoting properties.

### 3.2. SDA Prolongs the Lifespan of C. elegans by Regulating Mitochondrial and IIS Signaling Pathways, but Not DR

To further investigate the working mechanism for SDA’s antiaging effect in *C. elegans*, we analyzed several genetic factors that have been extensively studied for their involvement in regulating aging, DR, mitochondrial respiration signaling pathways, and IIS pathways in *C. elegans* [24].

DR is one of the most effective nondrug intervention methods for a broad variety of organisms, in addition to genetic editing [25,26,27]. There is a negative correlation between nematode lifespan and fecundity or brood size. In addition, longer lifespans are accompanied by lower reproductive rates [28]. However, contrary to what was expected, we observed that in the treated worms, the number of larvae did not decrease as the reproduction time increased. These results suggested that SDA could not promote life extension in a similar way to DR (Figure 3G). Using SDA in the development of antiaging drugs will not impair the reproductive capacity of *C. elegans*.

The mechanism of mitochondrial senescence theory is that oxidative damage to mitochondrial DNA leads to the impairment of mitochondrial structure and function, affecting the energy supply of cells; thus, a series of senescence symptoms occurs [29]. We found that SDA inhibited paraquat-induced ROS production (Figure 1D). ROS is a group of natural byproducts mainly produced in mitochondria, and the lifespan of mitochondrial mutant strains has been shown to correlate with ROS-mediated signaling and oxidative stress. The isp-1 and clk-1 encoded a catalytic subunit of mitochondrial complex III and ubiquinone biosynthesis, respectively. Results suggested that the effect of SDA on the lifespan extension might be partially dependent on isp-1 and clk genes. In short, SDA could extend the lifespan of *C. elegans* via mitochondrial respiration. 

DAF-16/FOXO plays a central role in the regulation of development, aging, and stress resistance in *C. elegans* [30,31,32]. It has been shown that the excessive intake of sugar leads to increased insulin levels, which will further inhibit *daf-16* activity and ultimately shorten the lifespan [33,34]. Moreover, it regulates aging, stress resistance, and immunity in *C. elegans*. It has been reported that *daf*-16 is responsible for the activation of major antioxidant machinery [35,36]. In the current study, we found that the lifespan-extension effect of SDA required *daf-16*. *Skn-1* activated enzymes that worked as radical scavengers and transferred glutathione [37]. *Skn-1* is located in the ASI neurons, in response to DR, or in the gut nuclei, in response to oxidative stress. Its accumulation in gut nuclei activates the phase 2 gene expression in response to stress, and is regulated by *sgk-1*, a serine/threonine kinase that can phosphorylate the *skn-1* transcription factor and modulate its nuclear localization [38]. The prolongation of nematode life by SDA may partly depend on the stress response molecule skn-1, which is also regulated by DAF-16 and IIS pathways. In *C. elegans*, the insulin-like receptor *daf-2* acts through PI3-kinase to activate the downstream of serine/threonine kinases *pdk-1*, *akt-1*, *akt-2*, and *sgk-1*, and subsequently regulates the *daf-16* activity [29]. Results showed an increase in the expression of *daf-2* (Figure 3K), which should have promoted a repression in the translocation of *daf-16* into the nuclei. In addition, nuclear translocation of *daf-16* in the fluorescent strain TJ356 was observed in worms treated with SDA (Figure 3I), distinctly. Our results showed that the *daf-16* mutant *daf-16(mu86)I.* totally suppressed the effects of SDA on lifespan extension and stress resistance, showing that the translocation of this transcription factor was essential to increase the lifespan of worms treated with SDA. The nuclear translocation of *daf-16* was a requisite for the transcriptional activation of target genes. We found that SDA did not further extend the lifespan of long-lived insulin-like receptor mutant *daf-2(e1370) III.*, mutant *akt-1(ok525) V.*, *akt-2(ok393) X.*, *sgk-1(ok538) X.*, or *pdk-1(sa680) X.*, but increased the expression of pathway-related genes. Moreover, SDA impacted the related upstream and downstream factors of DAF-16 in the IIS pathway.

### 3.3. Key Points of Experimental Operation

ROS in elegans is a highly active and short-lived molecule. It is almost impossible to detect it in vivo directly in biological samples. ROS levels can be measured indirectly using reporter probes. These molecular probes were designed to be absorbed by cells and emit fluorescent signals after being oxidized by ROS. The transparency of elegans makes them particularly suitable for the use of these molecular probes, allowing site-specific visualization of ROS formation. This is a significant advantage over fruit flies and mice [39]. Like many other fluorescent probes, H2DCFDA is photoactive, so it is important to minimize exposure to light during treatment. When measuring ROS in *C. elegans* cells with H2DCFDA, we used the whole nematode for testing to prevent the cleaved nematode from releasing intracellular metal ions and increase its own ROS, which had an impact on the results. Moreover, we measured the fluorescence intensity within 4 h after the start of the test to prevent H2DCFDA from easily diffusing into the cells.

As is known, ensuring the robustness of lifespan experiments can be challenging. Synchronization of cohorts is vital for elegans lifespan studies. In the lifespan experiment, we dissolved pregnant adults in bleach solution to euthanize the adults but not their eggs, so as to keep the lifespans of nematodes used in the experiment uniform. In lifespan studies, contaminations can be difficult to control, as old worms are likely to be killed if transferred repeatedly. Therefore, careful nematode-transfer techniques, aseptic techniques, and frequent inspection of inventory are necessary. The method we used to directly control the cell density was to measure the bacterial cell density by spectrophotometer with op50 overnight in Luria–Bertani liquid medium at 37 °C, and then concentrate the raw material to the required cell density (OD_600_ = 0.6) to keep the OP50 bacterial cell density consistent across plates and between experiments for the repeatability, and to prevent unexpected changes in food supply. Throughout the lifespan test, randomization and blinding also prevented other potential confounders. Any lifespan test required at least three independent, blind replications. Most importantly, when conducting nematode experiments, we recorded and reported as many details of relevant factors as possible in order to ensure the traceability and authenticity of the experimental results.

## 4. Materials and Methods

### 4.1. Reagents and C. elegans Strains

The SDA was obtained from the fruits of mangrove *S. apetala,* as we previously described [4]. All reagents were purchased from Beijing Solarbio Science & Technology Co., Ltd. (Beijing, China), and Nile Red and H2DCF-DA were obtained from Sigma-Aldrich Co., (St. Louis, MO, USA).

The Caenorhabditis Genetics Center (University of Minnesota, MN, USA) provided the *Escherichia coli* OP50 and *C. elegans* strains: CL4176 (*smg-1 (CC546) I*; *dvIs27X*) and TJ356 (*DAF-16::GFP(ZLS356*)); DA465 (*eat-2(ad465) II.*); CF1903 (*glp-1(e2144) III.*); CB4876 (*clk-1(e2519) III.*); ZG31 (*hif-1(ia4) V.*); RB1206 (*rsks-1(ok1255) III.*); EU1 (*skn-1(zu67) IV.*); TJ1052 (*age-1(hx546) II.*); JT9609 (*pdk-1(sa680)X.*); RB759 (*akt-1(ok525) V.*); VC204 (*akt-2(ok393) X.*); VC345 (*sgk-1(ok538) X.*).

### 4.2. C. elegans Culture

The strains used in this study were maintained on nematode growth media (NGM) plates at an appropriate temperature, as previously described [40]. The adult worms were dissolved in a bleach mixture (5 M NaOH, 10% NaClO) and then washed three times with M9 buffer. Next, the eggs were synchronized and transferred to the incubator to hatch into L1 larvae. Synchronized L1 larvae were cultured to L4 larvae for subsequent tests. SDA was dissolved in DMSO and diluted to the desired concentration prior to use. After the SDA solution was added to the NGM plate containing inactivated OP50 (65 °C for 30 min), the final DMSO concentration was 0.1%. The control group had the same solvent concentration as the SDA group [41].

### 4.3. Lifespan Assays

Lifespan assays were performed as described in a previous report [35]. Briefly, at least 60 L4 larvae were transferred to fresh NGM plates on day 0 of the assay. *C. elegans* were transferred to fresh plates supplemented with 100 μM SDA or 0.1% DMSO (vehicle control) on every other day. Worms were considered dead when they failed to respond to touch with a platinum wire. Moreover, worms were censored if they crawled off the plate, displayed extruded internal organs, or died because of hatching progeny inside the uterus [42]. The entire lifespan assay was repeated in at least three independent experiments.

### 4.4. Assay for Paraquat-Induced Oxidative Stress 

For the oxidative stress test, synchronized L4 wild-type larvae were transferred to NGM plates containing 5 mM paraquat at 20 °C on the first day. On the following days, nematodes were monitored and scored as dead when they did not respond to touch with a platinum-wire picker.

### 4.5. Measurement of Reactive Oxygen Species Production

To measure the reactive oxygen species (ROS) levels in *C. elegans*, at least 30 synchronized L4 wild-type larvae were cultured, and the procedure was the same as that for the oxidative stress resistance assay described above. After five days of feeding, worms were washed three times with M9 buffer to remove OP50 bacteria. Then, 50 μM H2DCF-DA (Sigma-Aldrich Co., St. Louis, MO, USA) was added to each NGM plate. After nematodes were incubated for 3 h at 20 °C, they were transferred onto microscope slides layered with 2% agar pads. The worms were anesthetized with 1% levamisole and then analyzed [43]. We quantified the fluorescence intensity at an excitation wavelength of 490 nm and an emission wavelength of 510–570 nm. We measured the fluorescence signal every hour and exported raw data. Blank-subtracted raw data (DCF fluorescence level) were normalized to those in untreated nematodes and were presented as mean standard deviation using Microsoft Office 2017 Excel software. A one-way ANOVA with a Tukey’s post hoc test was carried out using SPSS Statistics 21 to analyze the statistical significance of differences.

### 4.6. Lipid-Staining Assay by Nile Red

Nile red staining was conducted as described in a previous report [43]. First, OP50 bacteria in the original concentration of 0.5 mg/mL were diluted with the Nile red solution at a ratio of 1:250 (*v*/*v*). Then, at least 30 synchronized L1 larvae were transferred to the respective NGM plates and cultivated at 20 °C for 72 h. Thereafter, nematodes were transferred to three different plates according to different observation times (3/6/12 days). On the day of imaging, nematodes were fixed with 1% paraformaldehyde solution, stained with 1 mg/mL Nile red for 30 min under dark conditions, and washed three times with M9 buffer. The elegans with fixed Nile red staining were observed with a 40× objective lens under a Leica fluorescence microscope (DM4B), and 16-bit images were taken with a CCD camera (DFC7000T). Fluorescence intensity was defined using Image J (US National Institutes of Health, Bethesda, MD, USA).

### 4.7. Autofluorescence Assay

After the incubations of synchronized L1 wild-type larvae in the presence or absence of 100 μM SDA for 72 h at 20 °C, worms were treated for 3/6/12 days and washed off using M9 buffer, and the anesthetized nematodes were transferred to microscope slides for observation. Each group, including at least 30 nematodes, was photographed, and the accumulation of fluorescent autofluorescence was analyzed as described in a previous work [44]. The elegans with fixed Nile red staining were observed with a 40× objective lens under a Leica fluorescence microscope (DM4B), and 16-bit images were taken with a CCD camera (DFC7000T; excitation/emission 358 nm/461 nm). The fluorescence intensity was calculated semiquantitatively using ImageJ, and represented in terms of normalized values of corrected total cell fluorescence (CTCF) (CTCF = integrated density (area of selected cell mean fluorescence of background readings)).

### 4.8. Paralysis Assays

Adult CL4176 worms were transferred onto NGM plates to lay eggs. After 2 h, they were taken out and transferred to the medium added with SDA. After two days of culture at 16 °C, the worms were shifted to grow at 25 °C for 31 h, and then scoring was performed every hour. The worms were scored as “paralyzed” when they failed to respond to the stimulus after being gently touched with a platinum wire [2].

### 4.9. Pseudomonas Aeruginosa Infection Assay

Synchronized L1 larvae were transferred onto NGM plates seeded with OP50 and cultured at 20 °C. The plates for infection assay were seeded with P. aeruginosa and cultured overnight before use. Synchronized L4 larvae were transferred onto NGM plates and seeded with the pathogen and cultured at 25 °C in the presence or absence of 100 μM SDA. The number of dead worms was scored every day.

### 4.10. Reproduction

At least 10 synchronized L4 larvae were picked into NGM plates with SDA or without SDA, as described in the lifespan assay. Worms were transferred to the pretreated plates to lay eggs every day until the end of the fifth day of the experiment, and eggs were counted after incubation at 20 °C for 48 h [45].

### 4.11. Intracellular Localization of DAF-16::GFP

The transgenic strains TJ356 were used to detect the intracellular localization of the GFP-tagged transcription factors. Synchronized L4 larvae were transferred to the prepared NGM plates with or without 100 μM SDA and maintained for 1 h at 20 °C. The control-group and the SDA-treatment-group worms were separately placed on a microscope slide and mixed with 1% levamisole, and the cellular localization of DAF-16::GFP was detected [46].

### 4.12. RNA Extraction and Quantitative Polymerase Chain Reaction

About 2000 synchronized adult worms were transferred onto five NGM plates (6 cm diameter) with or without 100 μM SDA and cultured at 20 °C for 48 h. Total RNA was extracted using a RNAiso Plus (Takara Bio Inc., Dalian, China). and converted to cDNA using a high-capacity cDNA reverse transcription kit (Takara Bio Inc., Dalian, China). Quantitative reverse-transcriptase–polymerase chain reaction (qRT-PCR) was performed using a Power SYBR Green PCR Master Mix (Bio-Rad Laboratories, Inc., California, America) and the ABI 7500 system (Takara Bio Inc., Dalian, China). The relative expression levels of genes were determined using the 2−ΔΔCT method and normalized to the gene expression, and β-actin was used as an internal control. The *p*-values were calculated using a two-tailed *t*-test [47].

### 4.13. Western Blot

CL4176 worms were treated with the same approach as in the paralysis assays. After the L4 nematodes were transferred to 25 °C and cultured for 31 h, the control group and SDA group CL4176 *C. elegans* were washed off from the plates using M9 buffer. The isolated proteins were analyzed via Western blotting [2,48]. The concentration of extracted protein was quantified with a Bioepitope Bicinchoninic AcidProtein Assay Kit. Equal amounts of protein were loaded onto Tris-Tricine gels. Proteins were transferred to polyvinylidene fluoride membranes (Solarbio, Beijing, China). The primary antibodies used for immunoblotting were anti-b-amyloid (Solarbio, Beijing, China) diluted 1:500 and anti-β-actin (Solarbio, Beijing, China) diluted 1:1000. The secondary antibodies used were horseradish peroxidase goat antimouse (Solarbio, Beijing, China) and horseradish peroxidase goat antirabbit (Solarbio, Beijing, China) diluted 1:5000. The proteins on the polyvinylidene fluoride membrane were detected with an enhanced chemiluminescence reagent (Solarbio, Beijing, China).

### 4.14. Statistical Analysis

The statistical analysis and graphical presentation of data were performed using GraphPad Prism version 5. Analysis of variance and a nonpaired t-test were used to calculate statistical significance where applicable. Significant differences between the lifespan of treated and control worms were determined using the Kaplan–Meier survival assay, and *p*-values were calculated using the log-rank test. The results were plotted as mean ± standard error of the mean (SEM).

## 5. Conclusions

In this study, we demonstrated that SDA had antiaging activity in *C. elegans*. This effect could be mediated through modulating the mitochondrial and IIS pathways. Moreover, SDA had no effect on the reproductive ability of the worms treated, suggesting that it has potential in the development of antiaging drugs.

## Data Availability

The data presented in this study are available upon request from the corresponding author.

## Supplementary Materials

The following are available online at https://www.mdpi.com/article/10.3390/md20010059/s1, Figure S1: Representative images of ROS; Figure S2: Representative images of relative lipid; Figure S3: Representative images of autofluorescence; Table S1: Primer sequences of genes used in experiment.
