# Supplementary material for: Sonneradon A Extends Lifespan of Caenorhabditis elegans by Modulating Mitochondrial and IIS Signaling Pathways
Source: Mar Drugs. 2022 Jan 8;20(1):59. doi: 10.3390/md20010059 (PMC8778700; doi:10.3390/md20010059)
Supplement: Supplementary file 1 [file marinedrugs-20-00059-s001.zip › Supplementary Materialsú¿IDú║marinedrugs-1530147ú⌐.pdf]

Supplementary data

1. Supplementary figures.

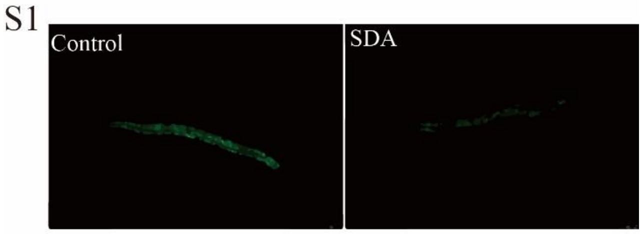

Figure S1. Representative images of ROS.

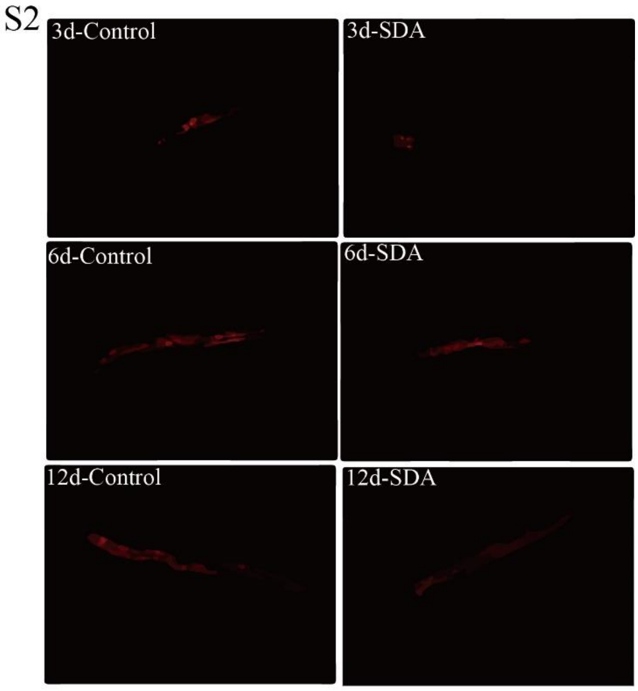

Figure S2. Representative images of relative lipid.

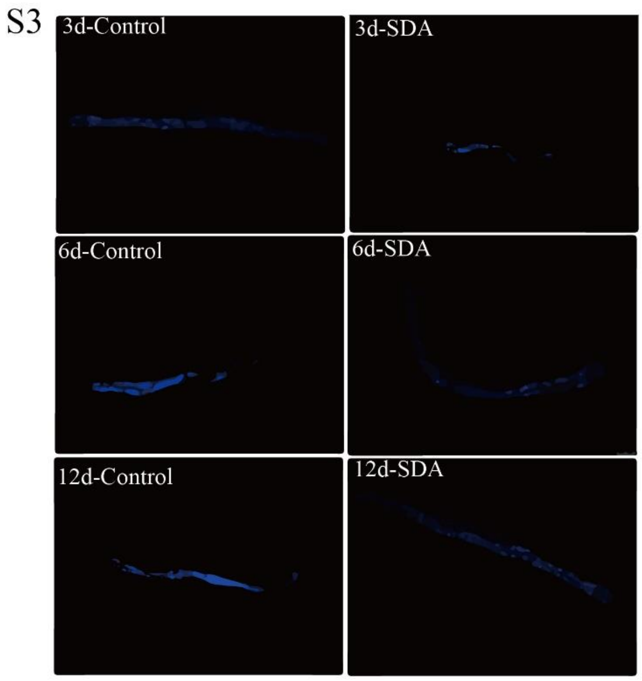

Figure S3. Representative images of relative autofluorescence.

Table S1. Primer sequences of genes used in experiment.

The annealing temperature is 55℃.

Gene	Type	Sequence
daf-16	F	5’- CCAGACGGAAGGCTTAAAACT -3’
	R	5’- ATTCGCATGAAACGAGAATG-3’
daf-2	F	5’- CGGTGCGAAGAGAGGATATT -3’
	R	5’- TACAGAGGTCGCCGTTACTG-3’
akt-1	F	5'-ATGCAGTTTGCGAATGGTGG -3’
	R	5'-AGCAAGAACGATCTCGGCTC-3’
akt-2	F	5'-ATTCAGCGAAGCACGAAC-3’
	R	5'-ATTCAGCGAAGCACGAAC-3’
sod-3	F	5’-AGCATCATGCCACCTACGTGA-3’
	R	5’-CACCACCATTGAATTTCAGCG-3’
ctl-1	F	5’-CGGATACCGTACTCGTGATGAT-3’
	R	5’-CCAAACAGCCACCCAATCA-3’
ctl-2	F	5’-ACACTCATTTCCACCGCCTT-3’
	R	5’-TCCCAGAATTGACGGGGTTG-3’

mtl-1	F	5’- GGAGGCCAGTGAGAAAAAATG -3’
	R	5’- GCTTCTGCTCTGCACAATGAC -3’
hsp-12.6	F	5’-GTGATGGCTGACGAAGGAAC-3’
	R	5’-GGGAGGAAGTTATGGGCTTC-3’
hsp-16.1	F	5’-GTCACTTTACCACTATTTCCGTCCAGCTCAACGTTC-3’
	R	5’-CAACGGGCGCTTGCTGAATTGGAATAGATCTTCC-3’
hsp-16.2	F	5’-CTGCAGAATCTCTCCATCTGAGTC-3’
	R	5’-AGATTCGAAGCAACTGCACC-3’
skn-1	F	5’-AGTGTCGGCGTTCCAGATTTC-3’
	R	5’-GTCGACGAATCTTGCGAATCA-3’
gst-4	F	5’- TCCGTCAATTCACTTCTTCCG -3’
	R	5’- AAGAAATCATCACGGGCTGG -3’
β-actin	F	5'-TCATGAAGTGTGACGTGGACATC-3’
	R	5'-CAGGAGGAGCAATGATCTTGATCT-3’
